# Benchmarking full-length transcript single cell mRNA sequencing protocols

**DOI:** 10.1186/s12864-022-09014-5

**Published:** 2022-12-29

**Authors:** Victoria Probst, Arman Simonyan, Felix Pacheco, Yuliu Guo, Finn Cilius Nielsen, Frederik Otzen Bagger

**Affiliations:** Genomic Medicine, Rigshospitalet, University of Copenhagen, Copenhagen, Denmark

**Keywords:** Benchmarking, Single cell, Full-length RNAseq, mRNA sequencing technologies, Takara, NEB, SMART-seq3, G&T sequencing

## Abstract

**Background:**

Single cell mRNA sequencing technologies have transformed our understanding of cellular heterogeneity and identity. For sensitive discovery or clinical marker estimation where high transcript capture per cell is needed only plate-based techniques currently offer sufficient resolution.

**Results:**

Here, we present a performance evaluation of four different plate-based scRNA-seq protocols. Our evaluation is aimed towards applications taxing high gene detection sensitivity, reproducibility between samples, and minimum hands-on time, as is required, for example, in clinical use. We included two commercial kits, NEBNext® Single Cell/ Low Input RNA Library Prep Kit (NEB®), SMART-seq® HT kit (Takara®), and the non-commercial protocols Genome & Transcriptome sequencing (G&T) and SMART-seq3 (SS3). G&T delivered the highest detection of genes per single cell. SS3 presented the highest gene detection per single cell at the lowest price. Takara® kit presented similar high gene detection per single cell, and high reproducibility between samples, but at the absolute highest price. NEB® delivered a lower detection of genes but remains an alternative to more expensive commercial kits.

**Conclusion:**

For the tested kits we found that ease-of-use came at higher prices. Takara can be selected for its ease-of-use to analyse a few samples, but we recommend the cheaper G&T-seq or SS3 for laboratories where a substantial sample flow can be expected.

**Supplementary Information:**

The online version contains supplementary material available at 10.1186/s12864-022-09014-5.

## Background

Within the last decade technologies for Single Cell Sequencing (SCS) has advanced research on tissue heterogeneity, cellular identity, and cellular state. Several initiatives applying single cell technologies at scale, including the Human Cell Atlas (HCA) project [1], Human Biomolecular Atlas Program (HuBMAP) from National Institute of Health (NIH) [2], and The LifeTime Initiative (https://lifetime-fetflagship.eu/) to mention a few. Single cell mRNA sequencing (scRNA-seq) allows for the study of inter- and intra-cellular transcriptional variability, and delineation of transient cellular processes, identification cell types, marker genes and pathways. All current scRNA-seq techniques require isolation and lysis of single cells with subsequent conversion of RNA to cDNA and amplification of cDNA. Amplification is necessary due to the small amount of starting material, limited to mRNA content in a single cell and current scRNA-seq protocols yields data that suffers from amplification bias [3]. Library preparation for scRNA-seq varies a lot in terms of what information it’s possible to uncover from the data, and the protocol should be carefully chosen depending on the biological problem at hand [4, 5].

**Fig. 1:**
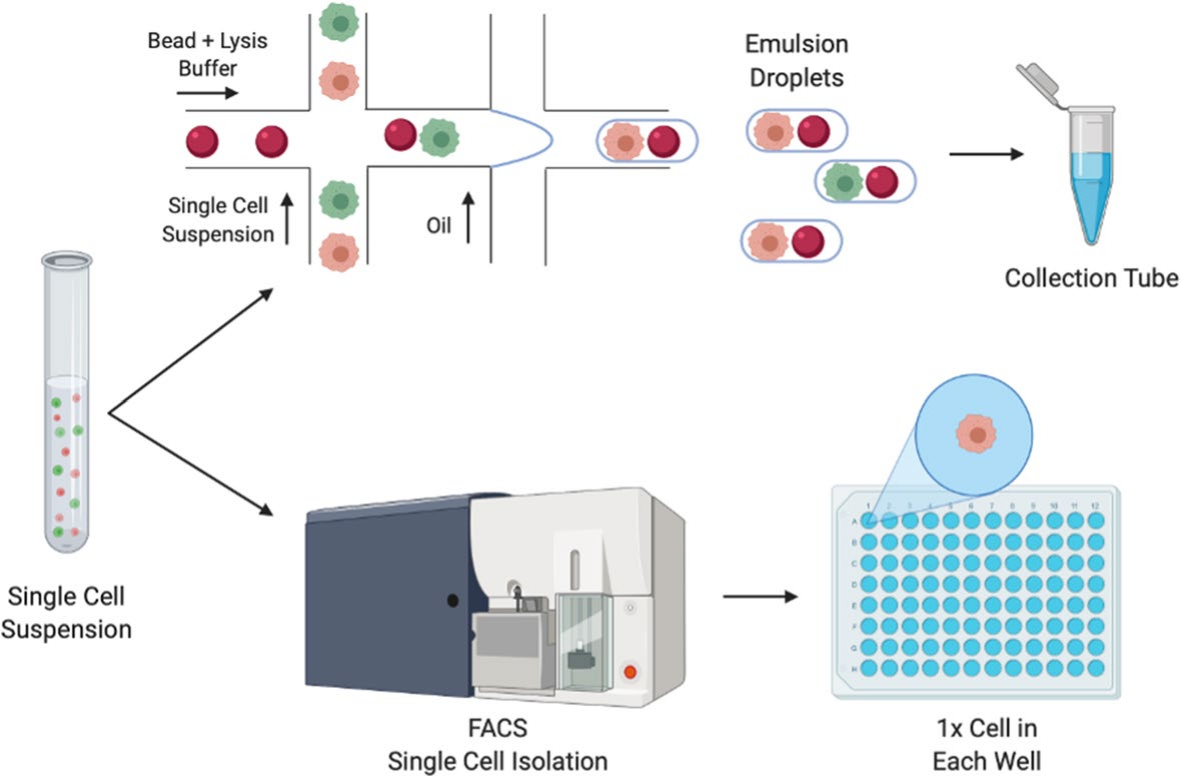
Workflow for droplet (top) and plate-based (bottom) single cell sequencing technologies. Droplet-based approaches combine primer-covered beads and single cells in emulsion droplets. In the droplets the single cell is lysed and reverse transcription (RT) is performed. Following RT, the synthesised cDNA is pooled and processed in bulk. In the Plate-based approaches, single cells are deposited in lysis-buffer filled chambers of a PCR plate or tube. Fluorescent activated cell sorting (FACS) may be used for this isolation of single cells

The *modus operandi* for single cell sequencing is the addition of a unique tag (barcode) to the DNA/RNA from each single cell, which in turn allows for highly multiplexed sequencing on a short-read sequencer, like popular machines from Illumina. After sequencing, demultiplexing allows for separation of data from each cell, using the barcodes. ScRNA-seq techniques are commonly distinguished in two categories: plate-based and droplet-based techniques. For both techniques the input material is single cells in suspension dissociated from e.g. tissue (Fig. 1). For the Plate-based techniques single cells are plated singularly into tubes or into each well of a PCR-plate. Barcodes are later added to each well as a step in amplification and final library preparation. For Droplet-based techniques, transcript barcoding takes place within the first step of the process, in a flow chamber making oil-emulsion droplets, with one cell per droplet. Recent commercial kits and flow chamber machines for droplet-based sequencing makes this protocol relatively easy entry but does not allow sequencing of the full-length transcripts. Plate-based methods require, in comparison, more technical know-how as well as separate handling of each cell. In practice plate-based methods only allow for processing of some hundreds of cells in parallel, whereas droplet-based methods can allow for the preparation of thousands of cells in a single batch. Plate-based approaches are still more sensitive and allow for detection of more genes per cell and furthermore allows for additional protocols on the same cell, such as quantification of FACS surface markers, and various DNA sequencing protocols [6–8] (Table 1).

**Table 1:** Table summarising *pro et contra* of droplet versus plate-based techniques

	**Droplet-based**	**Plate-based**
**Detected genes/per cell** [6]	200-5000	4000 ~ 8000
**Detected cells** [9]	1 k-10 k per run	1 cell per well
**Processing time** [9]	~ 9-10 h	~ 25 h
**Type of RNA analyzed**	3’ – or 5’end cDNA	Full length cDNA
**Others**	Require Immediate processing UMI-based	Enable collection months before processing

In order to uncover structural variation such as RNA fusions, mutations within transcripts, detection of pseudogenes, and splice variants, sequencing of the full-length transcript is needed. Full-length transcript scRNA-seq techniques are currently all plate-based. A disadvantage of conventional full-length sequencing techniques is the preclusion of early barcoding and incorporation of Unique Molecular Identifiers (UMIs). Adding UMIs in an experiment aims to establish a unique identity of each RNA molecule [10]. During PCR amplification, each cDNA containing the same UMI is assumed to be derived from the same mRNA molecule. Inclusion of UMI’s counting gives the protocol higher power with regards to transcript copy number detection [11, 12].

SMART-seq (Switching Mechanism At the 5' end of RNA Template) is a plate-based technique selectively capturing polyadenylated (poly(A)) RNA transcripts. The protocol yields libraries of full-length transcripts and relies on Reverse Transcription (RT) followed by template switching (TS) [13]. In brief, the poly(A)-tail of mRNA transcripts are primed using an oligo-d(T) primer coupled to a PCR handle. The primed mRNA is reverse transcribed by Moloney Murine Leukemia Virus (M-MLV) RT, which has terminal transferase activity, and adds non-templated nucleotides to the 3’end of cDNA ends. These non-templated nucleotides are preferentially cytosines, which allow annealing of a template switching oligo (TSO) containing ribo-guanosine at its 3’end. SMART-seq2 is the second generation of the previous and differs from the original by utilising a TSO carrying a locked nucleic acid (LNA) at the 3’end which locks the nucleotide in endo-formation. The effect is improved base-stacking and annealing ability which in turn results in a higher melting temperature between the TSO and the cDNA strand [14]. The LNA gives SMART-seq2 higher transcript capture, which results in improved sensitivity in gene detection [15]. SMART-seq2 has previously been reported to be the most sensitive and accurate method in terms of gene detection, and gives the most even read coverage, among current scRNA-seq protocols [5, 15]. Today SMART-seq kits are commercially available differing in chemistry, price, and hands-on processing time. In this study, three different SMART-seq full-length protocols; NEBnext® Single Cell/Low Input RNA Library Prep Kit for Illumina (New England Biolabs (NEB®)), SMART-seq® High-Throughput (HT) kit (Takara Bio Inc.), and G&T-seq were performed on T47D cell line for comparison of sensitivity and precision between each protocol.

### NEBnext® Single Cell/Low Input RNA Library Prep Kit for Illumina

**Fig. 2:**
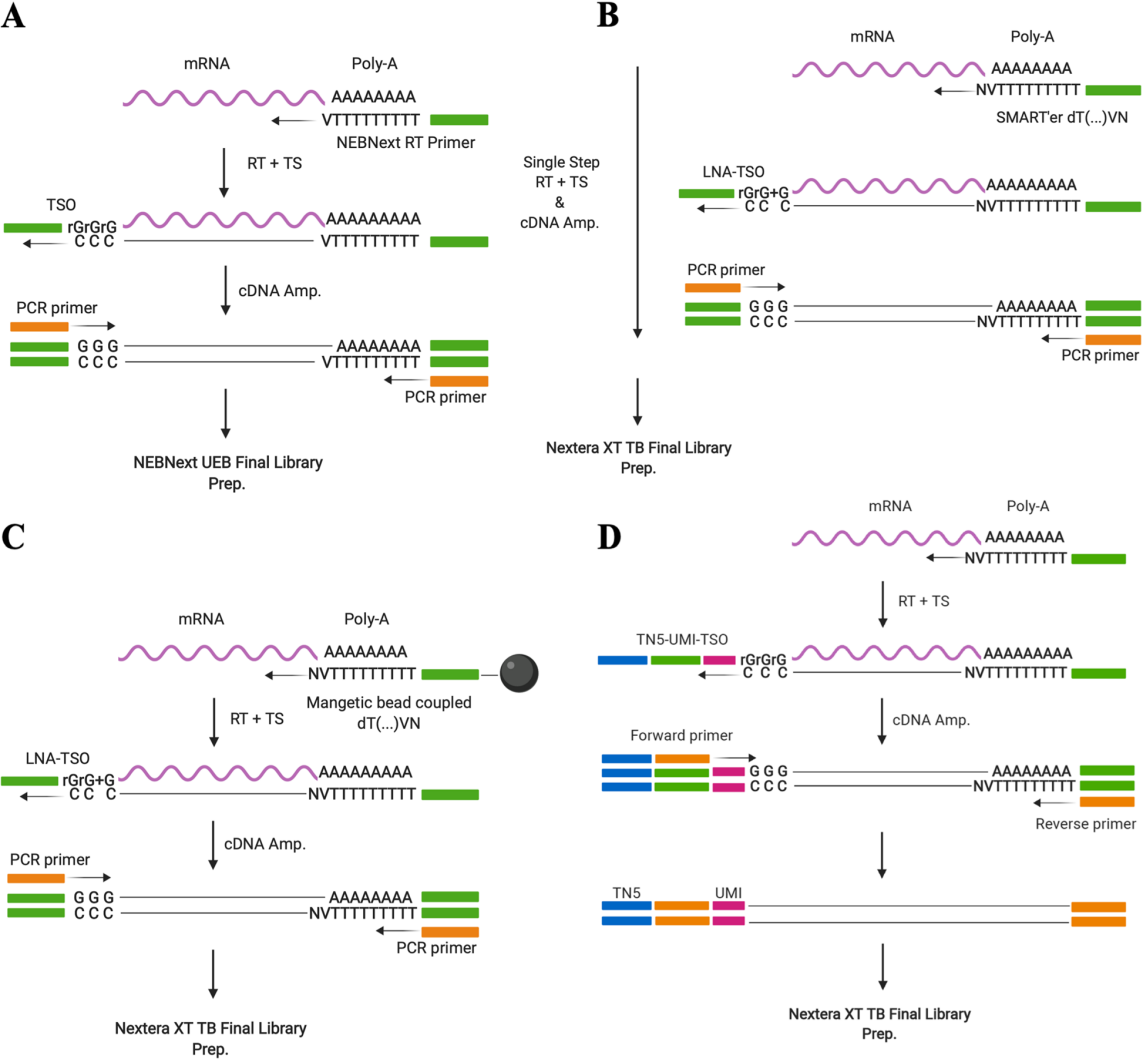
Illustration of the four scRNA-seq protocols applied in this study. **A** NEBNext® Single Cell/Low Input RNA Library Prep Kit for Illumina (NEB®) followed by NEBNext® uracil excision based (UEB) Final Library Preparation KIT. **B** SMART-seq® High-Throughput (HT) kit (Takara Bio Inc.) followed by final library preparation using Nextera XT Library preparation kit (Illumina, USA). **C** Genome & transcriptome sequencing (G&T-seq) followed by final library preparation using Nextera XT Library preparation kit (Illumina, USA). **D** SMART-seq3 (SS3) followed by final library preparation using Nextera XT Library preparation kit (Illumina, USA) TN5

NEB**®** is a commercially available kit containing enzymes and buffers required to convert RNA, either purified or from cultured or primary cells, to cDNA for sequencing on Illumina platforms. The kit is plate-based (or tube) and builds upon the techniques of original SMART-seq. NEB ULTRA II FS DNA library preparation for preparation of Illumina sequencing compatible libraries is included with the kit. The protocol had a price tag of 46 € per single cell when processing 12 reactions (Table 2). Advantageously, this kit includes both Reverse transcription (RT), PCR amplification, and final library preparation with no further reagents needed for sequence-ready libraries compatible with sequencing on Illumina machines (Fig. 2A). From here on, the protocol will be referred to as NEB®.

**Table 2:** Table constituting the number of cells processed and cells remaining post QC filtering

Protocol	NEB®	Takara®	G&T	SS3
**Cells Processed**	14	13	13	14
**Cells Post Filtering**	11	11	11	11
**Price per SC (12rxn)** **/ per 96rxn**	46 €^a^ / 4.024 €^a^	75 €^a^ / 5.955 €^a^	12 €^a^/ 1.152 €^a^	10 €^a^/ 960 €^a^
**Full Length**	**✓**	**✓**	**✓**	**✓**
**LNA**	✗	✓	✓	✗
**UMI**	✗	✗	✗	**✓**

All prices are per single cell using the smallest commercially available format of 12 cell reactions (rxn), as quoted medio 2020. All tested protocols feature full-length sequencing of transcripts. Takara® and G&T protocol genes a TSO with a locked Nucleic Acid (LNA), whereas NEB® and SS3 does not. Only SS3 features UMI’s

^a^Price includes reagents for reverse transcription, PCR amplification, and final library preparation for all protocols. Price for protocols Takara, G&T & SS3 include price for final library preparation at 8 € per cell for ¼ Nextera XT DNA library preparation (Cat. Nr: FC-131–1096) according to recommendation by kit manufacturer (*Takara Bio Inc.*) and protocol (G&T) [16]

### SMART-seq® HT kit

SMART-seq**®** HT kit (Takara Bio Inc.) is commercially available and designed for generating full-length cDNA from single cells or purified total RNA. The mechanism of the reaction is built upon a patented version of SMART-seq. SMART-seq® HT applies the newer SMART’er technology, SMART-seq2. The kit differs from traditional SMART-seq2 protocol by combining RT and cDNA amplification in a single step, which minimizes hands-on time. In this comparison, final library preparation was performed using Nextera XT Library preparation kit (Illumina, USA). This protocol is the most expensive at 73 € per single cell when processing 12 reactions (Table 2). This price includes RT and PCR amplification, as well as final library preparation according to recommendations by manufacturer (Fig. 2B). From here on, this protocol is referred to as Takara®.

### G&T Protocol

Genome & Transcriptome sequencing (G&T-seq) is not a commercially available protocol, and was originally developed for the study of both the genome and transcriptome of the same single cell [17]. G&T protocol has previously been shown to outperform traditional SMART-seq2 protocol [16, 18]. The key divergence of the original SMART-seq2 method, is a step separating mRNA from genomic DNA (gDNA), into distinct single cell samples eligible for a range of library preparation protocols. This step also serves as an RNA purifying step, removing cell debris, protein and gDNA from the downstream reaction, and the method has been applied to hard-to-sequence cells, where the DNA fraction has been discarded [19]. The protocol originally applied a modified SMART-seq2 protocol for transcriptome amplification, and PicoPLEX or Multiple displacement amplification (MDA) for genome amplification [13, 17]. Within the cell lysate, gDNA and mRNA is separated by the use of an oligonucleotide containing PCR sequence coupled to biotin at the 5’end. The oligonucleotide also contains a stretch of 30 thymidine residues (oligo-d(T)30) and an anchor sequence (VN)(V = A,G, or C; N = A,G,C or T). The function of the 5’Biotin modification is the ability to pair with streptavidin coated magnetic Dynabeads® (Fig. 2C). The mRNA transcripts are captured by the Oligo-d(T)30VN beads and a magnet used to move the complex to one side of the well. The lysate containing gDNA is subsequently transferred to a new plate. Following separation, gDNA and mRNA is individually processed and sequenced, allowing for correlation of genomic mutations with gene expression. The G&T-seq workflow is the second cheapest (12 € per single cell), however also the most demanding protocol to set up (Table 2). Each reagent has to be individually purchased and solutions prepared. The RT step also requires specialized equipment (Eppendorf Thermomixer C) for on-bead SMART-seq2 conduction. Throughout this article the protocol is referred to as G&T.

### SMART-seq3

SMART-seq3 is also a full-length scRNA-seq protocol with implemented UMI’s in the 5’end of full-length RNA transcripts [20]. The principle for adding UMIs is to establish a unique identity of each RNA molecule. During PCR amplification, each cDNA containing the same UMI will be considered derived from the same transcript molecule. Inclusion of UMI’s counting gives the protocol higher power in regard to CNV detection. It has been suggested that SMART-seq3 improve sensitivity of original SMART-seq protocols to a sensitivity level resembling single-molecule RNA fluorescence in situ hybridization (smRNA FISH). The protocol chemistry differs from previous generation SMART-seq by switching of the salt components from KCl to NaCl, utilisation of a next-generation MMLV RT, including 5% PEG during RT to improve cDNA yield, and finally by addition of GTPs during RT to support TS. This protocol has been reported to be more sensitive than SMART-seq2 protocol [20, 21]. In this comparison, final library preparation was performed using Nextera XT Library preparation kit (Illumina, USA). This protocol is the cheapest at 10 € per single cell when processing 12 reactions (Table 2). This price includes RT and PCR amplification, as well as final library preparation (Fig. 2D). From here on, this protocol is referred to as SS3.

### Quality measures for single cell RNA-seq

Common quality metrices applied in scRNA-seq are library size and nr. of genes detected per cell. Library size is the total sum of mapped sequencing reads (counts) across all genes for a single cell. Library size mainly depends on sequencing depth but given that an adequate number of reads have been obtained from sequencing, cells with small libraries can be considered of low quality. Small library size can be the result of RNA degradation due to either contamination (e.g. RNases), apoptosis, inefficient transcript capture before first strand synthesis or cDNA amplification. Importantly for our comparison, library size may also depend on protocol chemistry, reflecting the ability of adaptor-ligated fragments to anneal to oligos on the flow cell. Often of interest for scRNA-seq protocols is the ability to detect the vast repertoire of different gene transcripts within each single cell. The more genes a protocol is able to identify the more sensitive the protocol is evaluated. Here, we assess each protocol’s ability to detect endogenous mRNA transcripts of single cells.

To account for technical biases in a full-length RNA-seq protocol, External RNA Control Consortium spike-ins (ERCCs) can be added. ERCCs consist of 92 synthetic transcripts of bacterial origin that function to standardise sequencing experiments by adding an equal amount to each single cell reaction prior to processing steps. ERCCs show minimal sequence homology with endogenous eukaryotic transcripts, but feature a poly-A tail, have different GC-content and vary in lengths [22]. The total proportion of reads mapping to ERCC spike-ins can be used to assess the quality of input cells, because it can be assumed to be inversely proportional to good-quality fragments from the cell which are available for sequencing [3]. Applying ERCCs in a sequencing experiment can also be used to account for biases such as primer capture- efficiency, batch effects and absolute RNA content estimation, because the same amount and concentration is added to each sample. The proportion of mitochondrial (MT) mapping reads, can also be used as a QC metric for cell quality [3]; the reason being that transcripts within mitochondria are better protected both from leakage and degradation [3, 10]. The different mitochondrial genome consist of 37 genes, and captured MT genes can be assessed as an endogenous capture-efficiency control the same way synthetic ERCCs spike-ins are used.

## Results

### Data generation

**Fig. 3:**
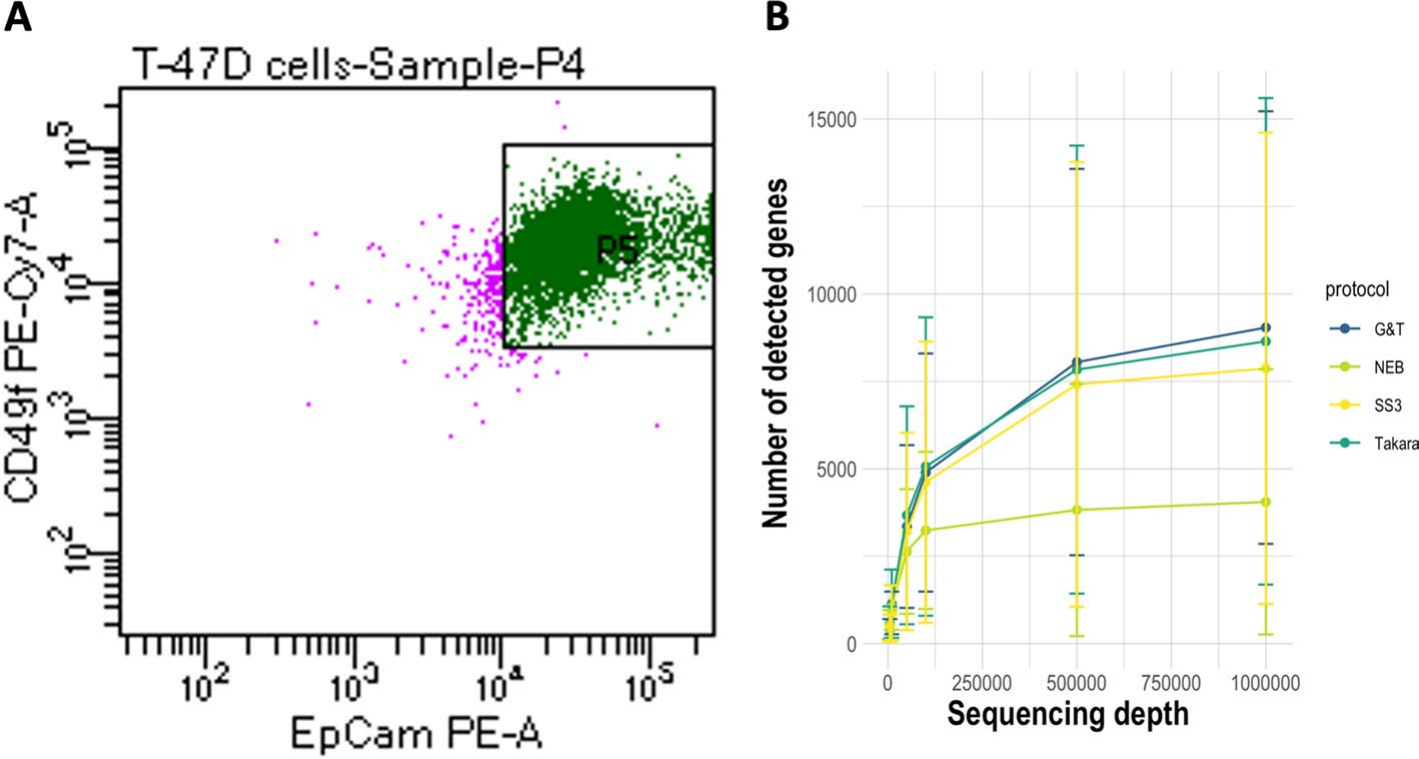
Cell Selection & Gene Saturation. **A** Homogenous EpCAM^+^/CD49f.^+^ T47D cells sorted into lysis buffer containing 96-well plates by fluorescent activated cell sorting. **B** Gene capture saturation prior to filtering performed by resampling subsets of total reads to 5000, 10.000, 50.000, 100.000, 500.000 and 1 million reads per cell using seqtk (https://github.com/lh3/seqtk)

Data was obtained by sequencing cells from breast cancer cell line T47D. mRNA from 13 single cells was amplified using Takara® SMART-seq® High-Throughput (HT) kit and G&T-seq protocol, while mRNA from 14 single cells was processed using NEB® NEBNext® Single Cell/Low Input RNA Library Prep Kit for Illumina and SMART-Seq3 protocol (Table 2). For retrieval of homogenous cell populations, cells were stained for, and selected by, expression of human EpCAM and Integrin α6 (CD49f) (Fig. 3A, Additional file 1: Fig. S1) [23, 24]. Cells were sorted by FACS into lysis buffer compatible with each protocol (Methods). Cells had same cell line origin and yielded similar results on measured parameters, and the major contributor to variability between single cells was cell cycle stage (Fig. 3A, Additional file 1: Fig. S2). We therefore consider each cell of same identity and use cells as biological replicates for benchmarking robust sequencing results. Single cells were sequenced in groups according to processing protocol on Miseq (Illumina, USA), aiming at a sequencing depth of one million reads for sufficient capture of genes across protocols (Fig. 3B) [5, 25, 26]. Following quality control (QC) and filtering, 11 cells from each protocol were kept in each dataset (Table 2). Processing of Cells and sequencing was performed uniformly for all tested protocols.

### Takara® had highest cDNA yield, NEB® had lowest

**Fig. 4:**
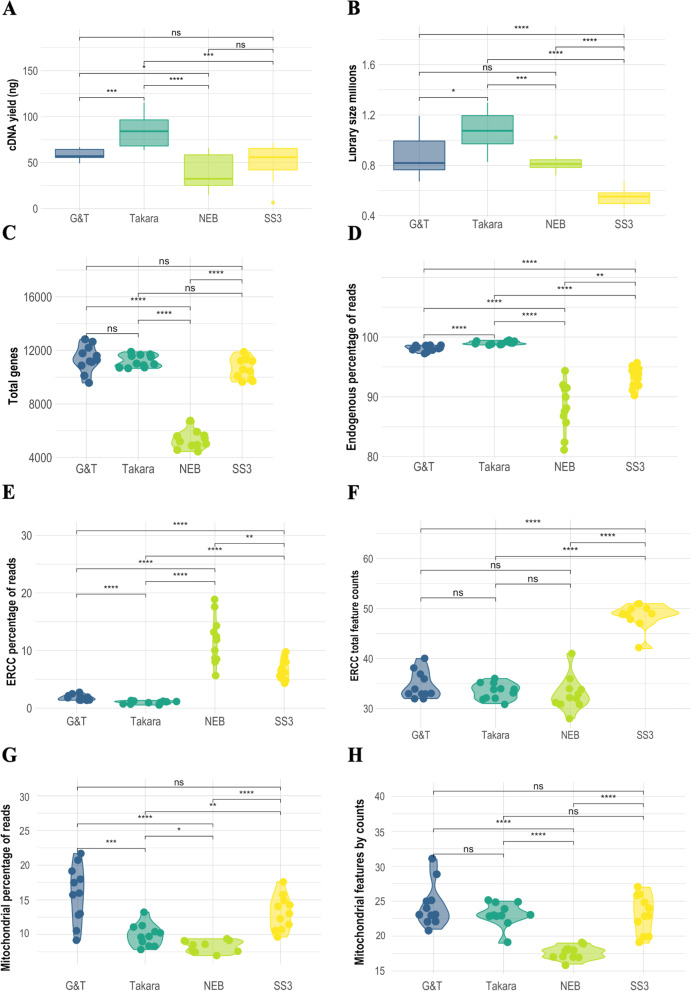
Comparative quality metrics from scRNA-seq data generated with NEB®, Takara®, G&T and SS3 protocols. **A** cDNA ng/yield following mRNA amplification in each protocol. **B** Library sizes millions. **C** Total nr. genes detected per single cell. **D** Percentage of reads mapping to endogenous genes per single cell. **E** Percentage of reads mapping to exogenous genes; ERCC spike ins per single cell. **F** Nr. Captured ERCC genes per single cell. **G** Percentage of reads mapped to mitochondrial (mt) genes per single cell. **H** Nr. Captured MT genes per singe cell. Each dot represents a single cell. Significance level cut-off: ns:*p* < 0.05, *:*p* <  = 0.05, **:*p* <  = 0.01, ***:*p* <  = 0.001, ****:*p* <  = 0.0001

In order to assess the RT and PCR amplification step, the total amount of cDNA was measured from each single cell library post PCR amplification. Takara®, G&T, and SS3 processed cells were subjected to 20 cycles of PCR amplification, whereas 22 cycles were necessary for successful amplification of NEB® processed cells. Cells processed using Takara®, and G&T had a total cDNA yield of ~ 84 ng (σ = 19 ng) and ~ 59 ng (σ = 7 ng) per single cell, respectively. (Fig. 4A). NEB® cells had the lowest average total cDNA yield of ~ 39 ng per single cell (σ = 20 ng) (Fig. 4A), despite the increased number of PCR cycles applied. SS3 had cDNA yield of ~ 48 ng per single cell (σ = 22 ng), not significantly different from NEB® (Fig. 4A).

### Takara® cells had largest libraries, SS3 had the smallest

**Fig. 5:**
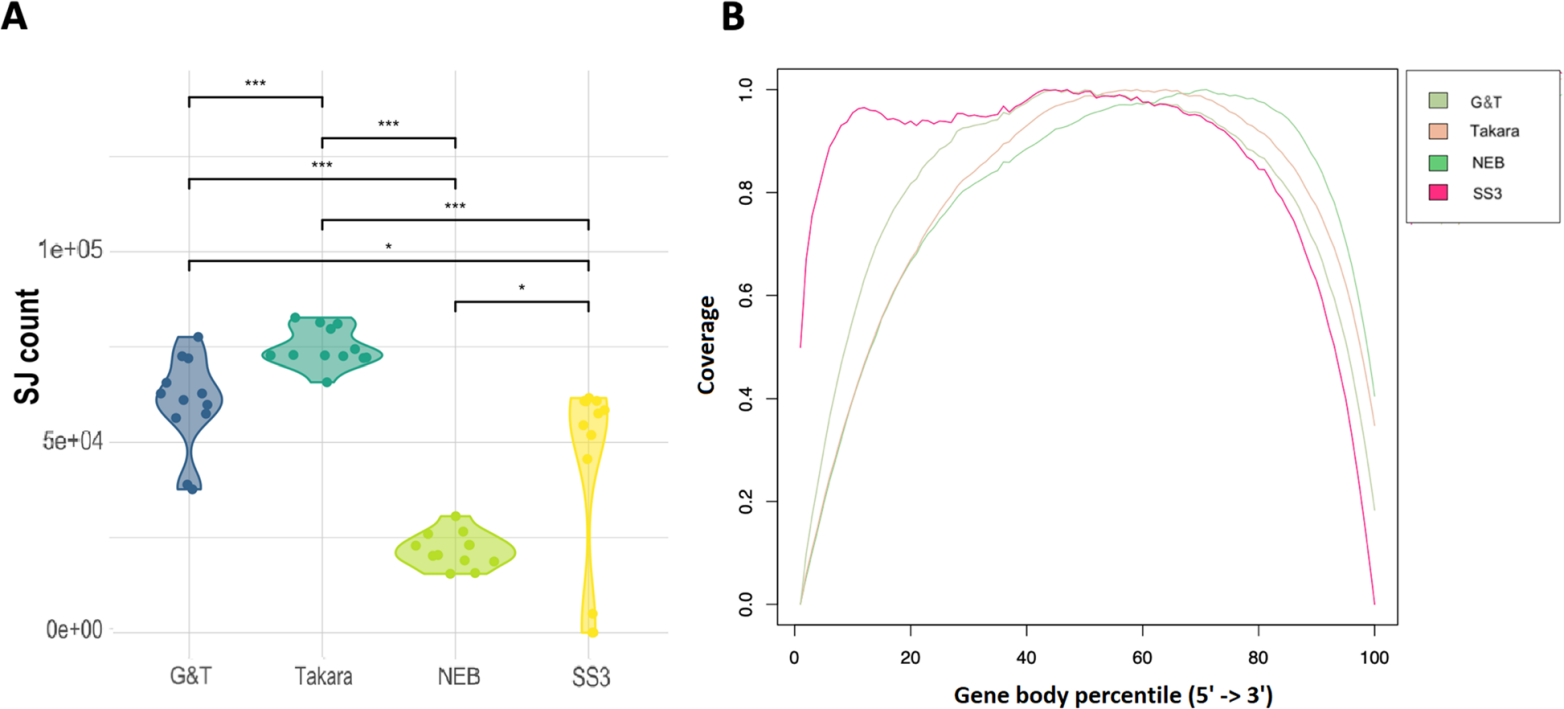
**A** Total number of Splice Junctions detected in the samples of each protocol. **B** Gene body coverage plot

In order to evaluate amplification efficiency, we assessed library size of each protocol. Single cell cDNA libraries where sequenced to the same depth and same sequencer, providing each protocol the possibility to obtain 1 million reads per single cell [5, 25, 26]. Given that sequenced cells are of equal type and quality, the capturing and amplification efficacy of the protocol reagents may lead to less than the targeted amount of reads (Fig. 4B, Table 3). Takara® cells had significantly (*p* < 0.02) larger libraries than remaining protocols with an average library size of 1.08 × 10^6^ reads per cell (σ = 0.2 × 10^6^ reads) (Fig. 4B, Table 3). G&T and NEB® cells had an average library size of 0.88 × 10^6^ reads per cell (σ = 0.2 × 10^6^ reads) and 0.82 × 10^6^ reads per cell (σ = 0.8 × 10^5^ reads), respectively (Fig. 4B, Table 3). However, NEB cells showed the most consistent library sizes yield across cells, between 0.72 – 1.02 × 10^6^ (Fig. 4B, Table 3). SS3 cells had the smallest libraries with an average of 0.55 × 10^6^ reads per cell (σ = 0.6 × 10^5^ reads) (Fig. 4B, Table 3), but also showed most gene body coverage bias, while G&T and Takara® showed least (Fig. 5B). The amount of genes with a coverage of at least 1x, 5x, 10x, and 100x, respectively (Fig. S6) reveals that SS3, G&T and Takara® produces a high coverage on many genes, and NEB produces a high coverage on few genes. Takara and G&T are consistently highest for each of the coverage thresholds.

**Table 3:** Table constituting summary of average library size, total genes captured, capture-efficiency of control genes

Protocol	NEB®	Takara®	G&T	SS3
Lib. Size (Millions)	0.82	1.06	0.88	0.55
sd	0.08	0.16	0.17	0.07
Total Features	5.310	11.211	11.382	10.677
sd	660	463	990	802
MT Features	18	23	24	23
sd	0.92	1.73	3.08	2.71
ERCC Feature	33	34	35	49
sd	3.38	1.57	2.69	2.5

MT and ERCC’s per single cell following filtering for protocols NEB®, Takara®, G&T, and SS3

*Sd* Standard error

### NEB® had the lowest capture-efficiency of control genes

Low fraction of reads mapping to ERCC and MT genes is a marker of high quality and robustness in single cell protocols [3]. Takara® cells had the lowest average proportion of reads mapped to ERCC spike-ins at 0.97% (σ = 0.26%), compared to G&T, SS3 and NEB® with 1.86%, 6.8%, and 11.75%, respectively. The ability to recapture ERCC was greatest for SS3 with an average of 48 out of 92 spike-ins, and similar for remaining protocols with 35 by both G&T and Takara®, 33 for NEB® (Fig. 4E F, Table 3). G&T, Takara®, NEB and SS3 cells had an average proportion of reads mapped to MT transcripts at 15.84% (σ = 1.6%), 9.91% (σ = 1.6%), 8.3% (σ = 0.84%), and 13.1% (σ = 2.5%) respectively. Average MT transcripts covered per cell for G&T, Takara®, and NEB was 24 (σ = 3.07), 23 (σ = 1.72), 18 (σ = 0.92), and 22 (σ = 2.7) transcripts per cell out of 37 MT genes. (Fig. 4G H, Table 3). The correlation between MT and ERCC may contribute to the understanding of the cell quality, in general the two should correlate equally among single cells. A higher fraction of ERCCs may indicate poor quality transcripts (eg. necrosis), whereas a higher fraction MT reads may indicate cell leakage. The correlation varied among protocols spanning from positive correlation (G&T: 0.9, Takara®: *ρ* = 0.33, SS3: *ρ* = 0.008) to negative correlation (NEB®: *ρ* =-0.64).

### G&T cells had the highest average gene count per single cell, NEB® cells had the lowest

In order to evaluate gene capture efficiency, the total number of genes with at least one read mapped was assessed in each single cell. Takara®, G&T, NEB and SS3 produced libraries with an average of 99% (σ = 0.26%), 98% (σ = 0.47%), 88% (σ = 4.09%) and 93% (σ = 1.75%) genes mapped to endogenous genes (Fig. 4D). Cells processed by G&T-seq recaptured the highest average number of 11.382 uniquely expressed genes per single cell (σ = 990). Takara® Kit and SS3 protocol produced cells with similar high gene detection at 11.211 genes (σ = 463) and 10.677 genes (σ = 802) respectively. NEB® kit had only half the coverage with an average of 5.310 genes per cell (σ = 661) (Fig. 4C, Table 3).

### Data from Takara® cells were most consistent over cells, while NEB® had the largest variance

**Fig. 6:**
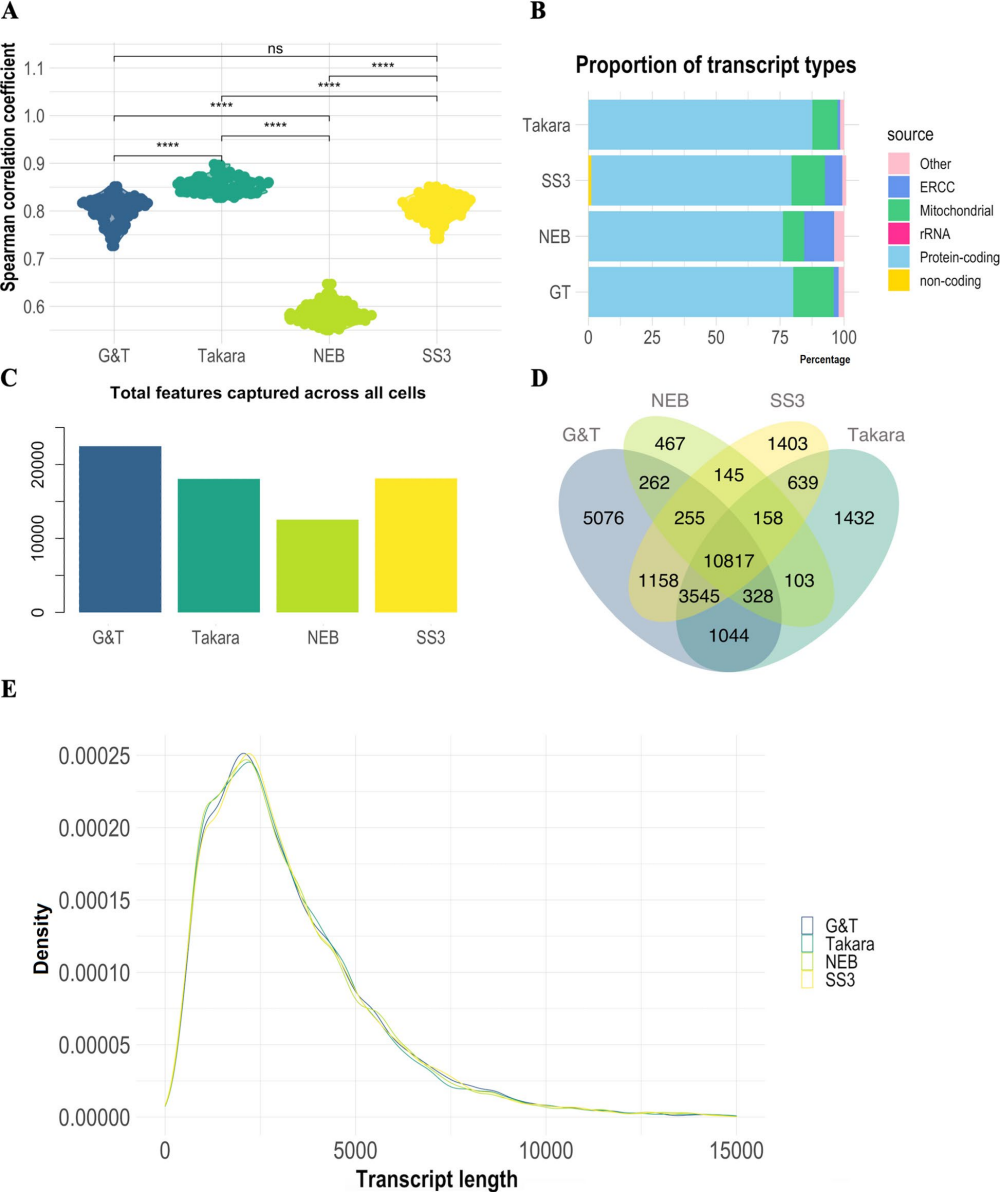
Comparative analysis of cell similarity, transcript type, length and unique genes between scRNA-seq data generated with NEB®, Takara®, G&T and SS3 protocols**. A** Spearman correlation coefficient (SCC) between all cells from the same protocol. **B** Proportion of different transcript types in each library. Non-coding transcript types constitute e.g. tRNA, snRNA, snoRNA, miRNA, miscRNA, lincRNA. **C** Bar-chart illustrating total unique genes captured across all cells by each protocol. **D** Venn diagram visualizing overlap of total captured genes between protocols, and genes captured uniquely in each protocol. **E** Average gene transcripts according to length (bp) across all tested protocols. Significance level cut-off: ns:*p* < 0.05, *:*p* <  = 0.05, **:*p* <  = 0.01, ***:*p* <  = 0.001, ****:*p* <  = 0.0001

In order to evaluate reproducibility between samples in each protocol, similarity between single cells was assessed by Spearman correlation coefficient (SCC) between all cells from the same protocol. NEB® processed cells were least similar with an average SCC of *ρ* = 0.52 (σ = 0.02) Takara®, G&T and SS3 cells showed significantly higher similarity between cells with an average SCC value of *ρ* = 0.82 (σ = 0.02), *ρ* = 0.74 (σ = 0.03) and *ρ* = 0.75 (σ = 0.03) respectively (*p* <  < 0.0001) (Fig. 6A). The same observation was made when subsetting the correlation geneset to the genes shared between protocols (Fig. S4).

### G&T cells captured the greatest number of unique genes across all cells

In order to evaluate the ability of each protocol to capture the vast repertoire of different transcripts, we assessed proportion of transcripts belonging to different biological categories, the total amount of genes captured by each protocol, as well as the amount of genes captured uniquely in each protocol (Fig. 6B C D). Proportion of captured transcript types were similar between all tested protocols (Fig. 6B, Fig. S3). However, Takara® had the largest proportion of transcripts mapping to protein-coding genes with an average of 88% (σ = 1.8%) compared to 80% (σ = 4%), 76% (σ = 3.6%) and 78% (σ = 2.5%) for G&T, NEB® and SS3 respectively (Fig. 6B). Takara also had the smallest proportion of unmapped transcripts whereas G&T had the highest (Fig. S3). G&T samples had the highest amount total genes captured across all cells at 22.485 genes (SS3: 18.120, Takara®: 18.066, NEB®: 12.535,) (Fig. 6C). Additionally, G&T cells had the largest number of genes which were uniquely captured by that protocol with 5.076 genes across all cells (SS3: 1.403, Takara®: 1.432, NEB®: 467) (Fig. 6D). The protocols differed most in the capture of genes related to peptide processing by the proteasome and mRNA processing (Fig. S5). G&T differed from SS3 and Takara® in capture of genes related to histone modifications and further from Takara® in capture of genes related to DNA replication (Fig. S5 B, E). Furthermore, Only G&T and Takara® did not differ in the capture of genes related to Golgi vesicular transportation (Fig. S5E). Finally, the distribution of gene transcript lengths reflects expected mammalian transcript distribution, suggesting that neither of the tested protocols had a transcript length bias and demonstrating that mRNA molecular quantification was not influenced on this measure in either of the protocols (Fig. 6E). Takara® and G&T processed cells also captured the most splice junctions, suggesting higher ability to study alternative splicing events between cells (Fig. 5A).

## Discussion

This study is a performance evaluation of four different plate-based scRNA-seq protocols; NEBNext® Single Cell/ Low Input RNA Library Prep Kit (NEB®), SMART-seq® HT kit (Takara®), Genome & transcriptome sequencing (G&T) and SMART-seq3 (Fig. 2). G&T protocol was found most sensitive in regards of gene detection, with the highest detection of genes per single cell (avg. 11.382 genes per cell) but not significantly different from Takara® and SS3 processed cells (avg. 11.211 (*p* = 0.48) & 10.677 (*p* = 0.15) genes per cell respectively) (Fig. 4C, Table 3). Furthermore, G&T captured the greatest number of genes across all single cells (22.284 genes), as well as having most genes uniquely captured by this protocol (5.076 genes) (Fig. 6C D). This suggest that G&T protocol might be superior in detecting transcripts across cells for improved unravelling of heterogeneity within a complex population of cells (e.g. tumor tissue). Takara® protocol showed a high gene detection similar to G&T protocol (avg. 11.211 genes per cell) (Fig. 4C, Table 3). Takara®, G&T and SS3 were found most consistent (SCC = 0.86, SCC = 0.80 and SCC = 0.81), suggesting high degree of reproducibility, especially important in e.g. a clinical setting (Fig. 6A). NEB® protocol was found least sensitive in regard to gene detection, both per single cell (avg. 5.310 genes per cell) and across all cells (12.535 genes) (Fig. 4C, Table 3). Low gene detection might lead to false positive detection of heterogeneity within a cell population, induced by undetected genes and not by true biology. In certain studies, as well as in diagnostics, quantification of single marker genes can be of great relevance, thus a high degree of undetected genes cannot be tolerated.

High correlation between ERCC and mtRNA reads for G&T (*ρ* = 0.9) would suggests robust quality metrics, whereas negative correlation in the case of NEB®(*ρ* = -0.64) could be a result of a competitive situation either during capture or sequencing. However, since the high performing Takara kit and SS3 protocol display little correlation (*ρ* = 0.33 and *ρ* = 0.008) between ERCC and mtRNA, we speculate that several quality metrices are needed to give a full picture of quality, as also suggested by *Ilicic *et al*. 2016.* Thus, results are likely affected by more than just competition for capture or reads. The technical differences between these protocols are believed to be caused partly by chemistry (e.g. LNA vs. no-LNA, lysis buffer, reverse transcriptase etc.) and reaction volume (lower volume = higher sensitivity) [18]. Only SS3 protocol is able to address PCR amplification biases, by implementing UMI’s in the 5’end of full-length RNA transcripts [21]. Inclusion of UMI counts may give the protocol higher power in regard to transcript copy-nr. detection [21]. Plate-based Quartz-seq2 and microfluidic 10xchromium 3’end RNA-seq are two examples of UMI featuring technologies that have previously performed well in regards to gene detection at low read depth [27]. However, the maximum nr. of captured genes remain at best one fourth lower for Quartz-seq2 and one fifth lower for 10xchromium 3’end RNA-seq, compared to G&T and Takara® protocols featured in this study [28–31]. 10xchromium 3’end RNA-seq technology, has also previously shown severe transcript drop-out risk, especially of rare transcripts [6]. Furthermore, both Quartz-seq2 and 10xchromium are not full-length protocols, limiting detection of analysis across all exons, fusion-transcripts, splice-variants, as well as SNP mutation analysis which are especially relevant in e.g. studies of disease [32–34]. 10xchromium 3’end RNA-seq allows parallel sequencing of up to ~ 80.000 cells in a single run; whereas, plate-based methods are limited most often to 96-well or 384-well format. Choosing protocol may therefore often boil down to the choice between high number of processed cells vs high number of detected genes.

**Fig. 7:**
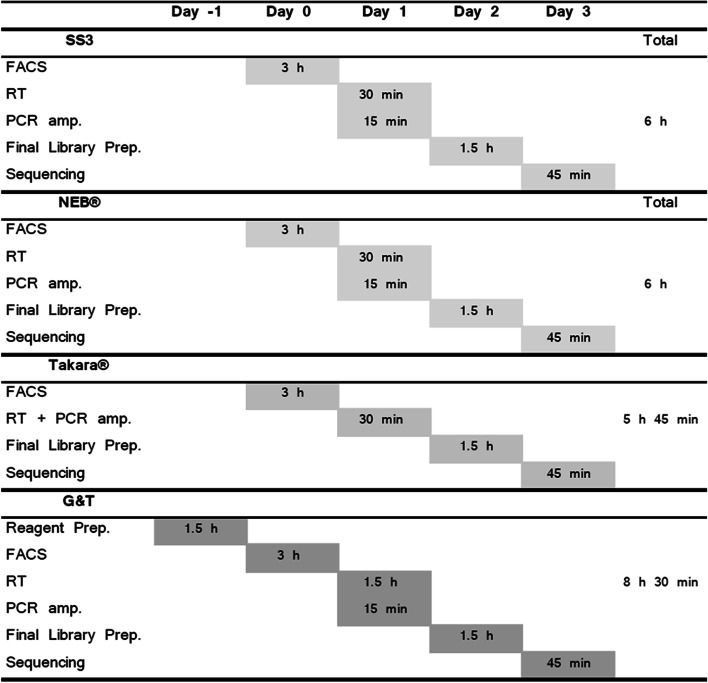
Hands-on time per protocol. Gantt chart featuring estimated hands-on time of each step of each tested protocol. NEB® and SS3 protocol has approximately 6 h of hands-on time. Takara® protocol has approximately 5 h and 45 min of hands-on time, by combining RT and PCR amp. Experimentalist save time both on processing but also leaves option for leaving the plate in thermocycler overnight, potentially saving even more time. G&T protocol has the longest processing time at 8 h 30 min due predominantly to reagent preparation and the separation step between RNA and DNA. All times are estimates assuming a skilled experimentalist with no prior experience with the protocol

Takara®’s SMART-seq® HT kit was evaluated as having the greatest ease-of-use, due to low hands-on-time, achieved by combining the step of reverse transcription and PCR amplification (Fig. 7). The ampure cDNA clean-up step of this protocol was also less time consuming and required fewer steps than NEB®’s Single Cell/ Low Input RNA Library Prep Kit. However, Takara®’s kit featured the highest total price per single cell (73 € Per cell/ sample). Furthermore, Takara®’s kit did not include reagents for final library preparation on Illumina machines and these must be purchased separately. NEB®’s kit included reagents for RT, PCR amplification, and final library preparation on Illumina machines. Even though NEB® did not outperform either G&T or Takara® on single cell metrices, this kit may perform well as a low input RNA-seq protocol—as a cheaper alternative to Takara®’s kit (46 € per cell/ sample). G&T protocol is not commercially available, and it is the second cheapest tested protocol (12 € per cell). G&T is also the most technically challenging to set up and requires some specialized equipment; the separation step of G&T-seq may be performed manually using a magnetic plate, but for more high throughput experiments, experimenters may wish to use a programmable liquid handling robot which could alleviate the manual burden. The RT step of G&T-seq furthermore requires a Thermomixer C (Eppendorf, cat. no. 5382 000.015), to prevent bead precipitation during on-bead RT reaction. The SS3 protocol is likewise not commercially available, and it is the cheapest tested protocol (10 € per cell). Technically SS3 is less requiring to set up than G&T-seq, however the gains of SS3 may be more challenging computationally than remaining protocols.

## Conclusions

Our comparison found that G&T processed cells showed the highest sensitivity in gene detection and high reproducibility at the lowest price. However, G&T was the most time-consuming and most technically challenging protocol. Takara® processed cells showed a likewise high sensitivity in gene detection similar to G&T processed cells, however at the absolute highest price. Takara® protocol had the greatest ease-of-use, lowest hands-on-time, as well as highest reproducibility across single cells. SS3 showed likewise high gene detection, high reproducibility at low price at entry-level technical requirements. NEB® cells showed lowest sensitivity of gene detection, and lowest degree of reproducibility between single cells. However, NEB® protocol had the advantage of including reagents for both RT, PCR amplification and final library preparation. In conclusion, we would recommend anyone with the skills and patience to perform G&T-seq due to the high sensitivity and reproducibility at a low price. If you are new to the field, Takara® offers a lower entry level protocol with high gene detection and high reproducibility across single cells, however at a higher price.

## Materials & methods

### Single cell suspension

T-47D single cell suspension was prepared by removal of growth medium and subsequent washing of cell layer using 10 ml PBS. Cells were disaggregated with 2 ml TrypLETM Express Enzyme (1X) (cat nr. 12,604,013, GibcoTM, USA) for approximately 2 min in an incubator at 37 C°. Reaction was stopped adding full growth media (RPMI-1640 + GlutaMAXTM (cat nr. 61,870–010, GibcoTM, UK) + 10% Fetal Bovine Serum (FBS) + 5% Penicillin/ Streptamycin) in double the amount of TrypLETM. Suspension was spun down 3 min 1200 rpm. Cells were washed once in PBS, resuspended in FACS buffer (PBS + 0.04% bovine serum albumin (BSA)), and filtered using a 100 μm strainer.

### Cell surface marker staining

Cell suspension was stained with monoclonal antibodies toward human EpCAM (cat nr. 130- 111–116, lot nr. 5,190,125,111, Miltenyi Biotec, Germany) conjugated to R-phycoerythrin (PE) (Ex-Max 496 nm/ Em-Max 579 nm), and Human Integrin α6 (CD49f) (cat nr. 25–0495-80, lot nr. 4,319,156, eBioscienceTM, USA) conjugated to PE-cyanine 7 (PE/Cy7, Ex-Max 496 nm/ Em-Max 785 nm). 4’,6-diamidino-2-phenylindole (DAPI) (Ex-Max 358 nm/ Em-Max 461 nm)(cat nr. 10,236,276,001, Roche Diagnostics GmbH, Germany) was used to discriminate dead from live cells. Concentration was adjusted to 105–106 cells/ml, and antibody added at concentration of 1 μl per 105–106 cells. Cells were incubated 15 min at 4 C° in the dark. Following incubation, dye was diluted, adding 1 ml of FACS buffer. Suspension was subsequently washed once with 1 ml FACS buffer. Pellet was resuspended in 1 ml FACS buffer. 10 μl DAPI was added per 1 ml cell suspension, to a final concentration of 300 nM, 5 min prior to sorting.

### Single cell sorting

Fluorescence activated cell sorting (FACS) was used for isolation of single T47D cells into lysis buffer containing 96-well semi-skirted PCR plates using instrument BD FACSAria™ III (BD bioscience, USA). Controls for calibrating instrument included unstained cells, cells stained with DAPI, and beads compensating for spectral overlap between fluorochromes PE and PE-CY7, using MACS Comp Bead Kits Anti-REA (cat nr. 130–104-693, lot nr. 5,181,012,289, Miltenyi Biotec, Germany) and Anti-RAT (cat nr. 130–107-755, lot nr. 5,181,015,526, Miltenyi Biotec, Germany). Cell suspension was gated to isolate double positive (EpCAM + /CD49f +) single T47D cells. A Multi-cell positive control (50 cells) and an empty-well negative control (0 cells), and at least one RNA-control diluted to 10 pg/ul were provided per plate. Following sorting cells were thoroughly vortexed, spinned down 1 min, flash- frozen in dry-ice and subsequently stored at -80 C°.

### NEBNext® Single Cell/ Low Input RNA Library Prep Kit

Fourteen single cells were processed using NEBNext® Single Cell/ Low Input RNA Library Prep Kit (cat nr. E6420S, New England Biolabs (NEB), USA), and were sorted into 5 μl NEBNext Cell Lysis buffer (0.5 μl NEBNext Cell Lysis Buffer (10x), 0.25 μl Murine RNase Inhibitor, 4.25 μl H_2_O). Protocol was performed according to recommendation by manufacturer with minor changes—1 μl 1:10^6^ dilution of ERCC spike ins (cat nr. 4,456,740, Invitrogen, Thermo Fischer Scientific, Lithuania) were added each single cell lysate prior to RT and PCR amplification was performed applying 22 cycles.

### SMART-seq® HT Kit

Thirteen single cells were processed using SMART-seq® HT kit (cat nr. 634,862, Takara Bio Inc, USA), and were sorted into 12.5 μl FACS dispensing solution (0.95 μl 10xLysis buffer, 0.05 μl RNase Inhibitor,1 μl 3’SMART-Seq CDS Primer II A, 10.5 μl Nuclease-free H2O). Protocol was performed according to recommendation by manufacturer with minor changes—1 μl 1:10^6^ dilution of ERCC spike ins (cat nr. 4,456,740, Invitrogen, Thermo Fischer Scientific, Lithuania) were added each single cell lysate prior to RT, and PCR amplification was performed applying 20 cycles.

### G&T-seq

Thirteen single cells were processed by G&T-seq and were sorted into 2.5 μl RLT Plus buffer (cat nr. 1,053,393, Qiagen, Germany). ScRNA-seq was performed as described by *Macaulay *et al*., 2016.* Single cell DNA sequencing featured in this protocol was not conducted in this experiment, but DNA stored at -80 C°. Each single cell lysate were added 1 μl 1:10^6^ dilution of ERCC spike ins (cat nr. 4,456,740, Invitrogen, Thermo Fischer Scientific, Lithuania) prior to RT. PCR amplification was performed applying 20 cycles.

### SS3

Fourteen single cells were processed by SS3 protocol and sorted into 3 μl SS3 lysis buffer mix [35]. ScRNA-seq was performed as described by *Sandberg *et al*. 2020*. Each well was added a concentration of 1:10^6^ ERCC spike ins (cat nr. 4,456,740, Invitrogen, Thermo Fischer Scientific, Lithuania). PCR mix was prepared using a working concentration of Kapa HiFi HotStart ReadyMix (1X) (Kapa, cat. no. KK2601), Fwrd. primer (0.5uM), Rev. primer (0.1uM). PCR amplification was performed applying 20 cycles.

### Sequencing

T47D single cell cDNA libraries were paired-end sequenced in groups according to each library preparation protocol (13 or 14 single cells per run) on MiSeq Benchtop Sequencer (Illumina, USA), using MiSeq Reagent kit v2 300 cycles (cat nr. MS-102–2002, Illumina, USA). Prior to sequencing each single cell library was diluted to a concentration of 4 nM in EB buffer + 0.1% Tween 20. Prior to sequencing 3 μl of each 4 nM library was pooled in an Eppendorf tube. 5 μl 4 nM pool was mixed with 5 μl 0.2 nM NaOH and incubated 5 min at RT, for denaturing of double stranded cDNA. The denatured sample pool was diluted to a concentration of 20 pM by mixing 10 μl 2 nM sample pool with 990 cold Hybridization Buffer 1 (HT1). Finally, 20 pM sample pool was diluted to a concentration of 10 pM, by mixing 500 μl 20 pM sample pool with 500 μl cold HT1.

### Alignment/ trimming

Illumina sequencing raw reads were converted to fastq files. Fastq files were processed on a bash shell. Raw reads were trimmed using Trim Galore v.0.4.0 [36] with default parameters, where two rounds of trimming were performed. The first trimming removed Nextera XT adaptors (”CTGTCTCTTATACACATCT”), and the second trimming removed cDNA amplification adaptors (”AAGCAGTGGTATCAACGCAGAGT”). Quality assessment of sequencing output was performed by two rounds of FastQC after each trim. Trimmed sequences were aligned using the splice-aware aligner STAR v2.5.2b [37] to Genome Reference Consortium Human Build 38 (GRCh38), with the following parameters: –genomeLoad NoSharedMemory –quantMode TranscriptomeSAM GeneCounts –readFilesCommand zcat –outSAMtype BAM SortedByCoordinate –limitBAMsortRAM 35,000,000,000. STAR output files comprising ReadsPerGene.out.tab for each sample, which were merged into a single tab delimited file, so called “count matrix”, for further analysis.

### Data visualisation

Introductory figures Illustrated using *Biorender* (https://biorender.com/). ScRNA-seq data was imported into R studies as an expression matrix. The count matrix was transformed into a Single Cell Experiment Object (SCE-object) using Rstudios (v3.6.1 Opensource, https://rstudio.com/) package *SingleCellExperiment* (v1*.*6.0). Data graphs were generated using *ggplot2* (v3.3.0). Plots are either Sinaplots [38] or box-plots where with outlier threshold at X and wiskers at Y, stars are denoted based on Wilcoxon test *p*-value; ns: p > 0.05, *: *p* <  = 0.05, **: p < 0.01, ***: p < 0.001, ****: *p* < 0.0001.

### Single cell Quality Control (QC)

Expression level of genes were quantified by CPM (counts per million). Genes with an average expression above zero (CPM > 0) across all cells were kept in the dataset. Cells not expressed in any cell (CPM = 0) were filtered away. Bad quality cells (or empty wells) were filtered away based on the following criteria: 1) cells that had less than 1000 uniquely expressed genes, 2) cells that had library sizes below 0.6e^6^ million reads, 3) cells that had more than 30% reads mapped to mitochondrial genes, and 4) cells that had more than 25% of reads mapped to ERCC spike-inn genes. Data analysis was performed with RStudio (version 3.6.1) using Bioconductor [https://www.bioconductor.org] packages (SingleCellExperiment, sinaplot [38], scater [39], ggplot2, GenomicFeatures [40], sincell [41], TxDb.Hsapiens.UCSC.hg19.knownGene, SummarizedExperiment, robCompositions, splatter [42], reshape2, ggforce, gdata, hrbrthemes, viridis, VennDiagram, DESeq2 [43], dplyr, tidyverse [44], gtable, gridExtra, hrbrthemes, ggforce, ggpubr) following guidelines from [https://scrnaseq-course.cog.sanger.ac.uk/website/index.html].

### Gene body coverage

Read coverage over gene body was analysed using geneBody_coverage method in RSeQC package v4.0.0 [45]. The further visualisation was performed using ggplot2 (v3.3.0) in R.

## Supplementary Information

**Additional file 1: Fig. S1.** Fluorescent activated cell sorting (FACS) of single T47D cells. Cells were stained for EPCAM-PE, CD49f-PE/Cy7, CD31-APC, CD45-FITCH. **Fig. S2.** Cell cycle distribution between single cells of protocol Takara®, G&T, NEB®, and SS3. Cell cycle assignment performed using cyclone (Scialdone et al., 2015). **Fig. S3.** Average proportion of Multimapped, No Feature, Ambiguous and Unmapped transcripts for each protocol. Significance level cut-off: ns:*p*<0.05. **Fig. S4.** Spearman correlation coefficient (SCC) between all cells from the same protocol. The correlation was made from a geneset of the 10817 shared genes shared between the protocols (Fig. 5 D). **Fig. S5.** Pathway enrichment analyses of genes captured differently between cells processed by protocol A) G&T and NEB®, B) G&T and SS3, C) Takara® and NEB®, D) SS3 and NEB®, E) G&T and Takara® and F) Takara® and SS3. KEGG [46] pathway enrichment analysis was performed and visualized by using clusterProfiler (v4.4.4) [47] package from R. **Fig. S6.** Calculations of coverage depth. A) Count of genes that have more than 1X coverage across coding regions.  B) Count of genes that have more than 5X coverage across coding regions.  C) Count of genes that have more than 10X coverage across coding regions D) Count of genes that have more than 100X coverage across coding regions.

## Data Availability

Data analyzed in this paper is publicly available from GEO accession number GSE155506.

## Abbreviations

NEB®: NEBNext® Single Cell/ Low Input RNA Library Prep Kit
Takara®: SMART-seq® HT kit
G&T: Genome & Transcriptome sequencing
SS3: SMART-seq3
SCS: Single Cell Sequencing
HCA: Human Cell Atlas
HuBMAP: Human Biomolecular Atlas Program
NIH: National Institute of Health
scRNA-seq: Single cell mRNA sequencing
FACS: Fluorescent activated cell sorting
UMIs: Unique Molecular Identifiers
SMART-seq HT: Switching Mechanism At the 5' end of RNA Template-sequence High-Throughput
RT: Reverse Transcription
TS: Template Switching
M-MLV: Moloney Murine Leukemia Virus
TSO: Template Switching Oligo
LNA: Locked Nucleic Acid
gDNA: Genomic DNA
MDA: Multiple displacement amplification
smRNA FISH: Single-molecule RNA fluorescence in situ hybridization
ERCCs: External RNA Control Consortium spike-ins
MT: Mitochondrial
QC: Quality Control
SCC: Spearman Correlation Coefficient
FBS: Fetal Bovine Serum
BSA: Bovine serum albumin
PE: R-phycoerythrin
DAPI: 4’,6-Diamidino-2-phenylindole
HT1: Hybridization Buffer 1
GRCh38: Genome Reference Consortium Human Build 38
SCE-object: Single Cell Experiment Object
CPM: Counts per million
