# Supplementary material for: Benchmarking full-length transcript single cell mRNA sequencing protocols
Source: BMC Genomics. 2022 Dec 29;23:860. doi: 10.1186/s12864-022-09014-5 (PMC9801581; doi:10.1186/s12864-022-09014-5)
Supplement: Supplementary file 1 — Additional file 1: Fig. S1. Fluorescent activated cell sorting (FACS) of single T47D cells. Cells were stained for EPCAM-PE, CD49f-PE/Cy7, CD31-APC, CD45-FITCH. Fig. S2. Cell cycle distribution between single cells of protocol Takara®, G&T, NEB®, and SS3. Cell cycle assignment performed using cyclone (Scialdone et al., 2015). Fig. S3. Average proportion of Multimapped, No Feature, Ambiguous and Unmapped transcripts for each protocol. Significance level cut-off: ns:p<0.05. Fig. S4. Spearman correlation coefficient (SCC) between all cells from the same protocol. The correlation was made from a geneset of the 10817 shared genes shared between the protocols (Fig. 5 D). Fig. S5. Pathway enrichment analyses of genes captured differently between cells processed by protocol A) G&T and NEB®, B) G&T and SS3, C) Takara® and NEB®, D) SS3 and NEB®, E) G&T and Takara® and F) Takara® and SS3. KEGG [46] pathway enrichment analysis was performed and visualized by using clusterProfiler (v4.4.4) [47] package from R. Fig. S6. Calculations of coverage depth. A) Count of genes that have more than 1X coverage across coding regions. B) Count of genes that have more than 5X coverage across coding regions. C) Count of genes that have more than 10X coverage across coding regions D) Count of genes that have more than 100X coverage across coding regions. [file 12864_2022_9014_MOESM1_ESM.docx]

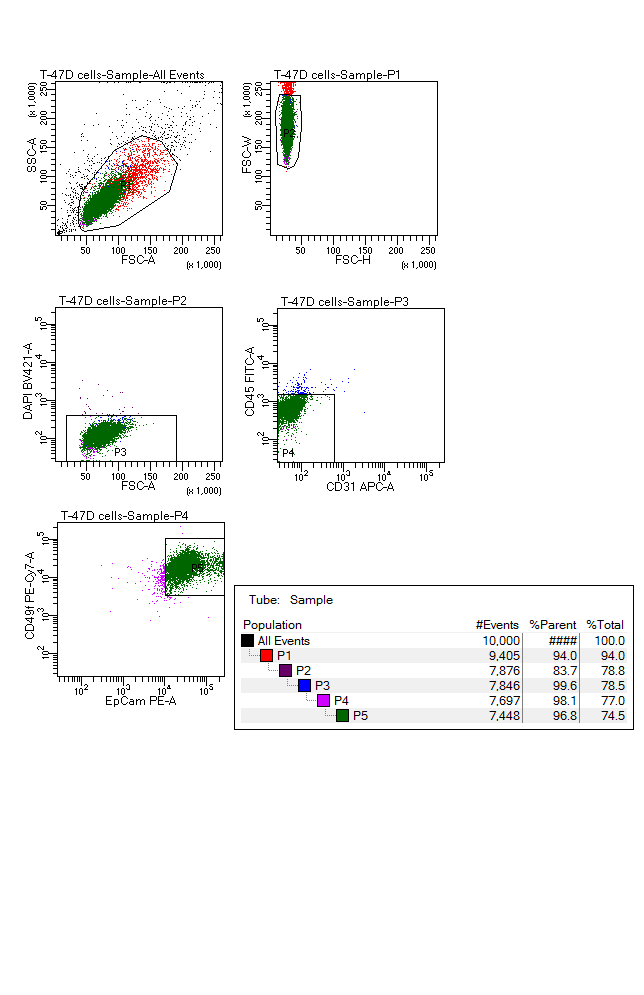
 **Fig. S1**

Fluorescent activated cell sorting (FACS) of single T47D cells. Cells were stained for EPCAM-PE, CD49f-PE/Cy7, CD31-APC, CD45-FITCH. P5: Double positive EPCAM+/CD49f+, sorted into lysis buffer containing 96-well plates according to protocol. P1: Population of live single T47D cells. P2: Singlet T47D cells, discarding doublets. P3: DAPI negative T47D cells, discarding dead/dying cells. P4: CD45-/CD31 negative population, control of non-specific binding of antibodies towards immune and endothelial cells.

**Fig. S2**

Cell cycle distribution between single cells of protocol Takara®, G&T, NEB®, and SS3. Cell cycle assignment performed using cyclone (Scialdone et al., 2015).

**Fig. S3**
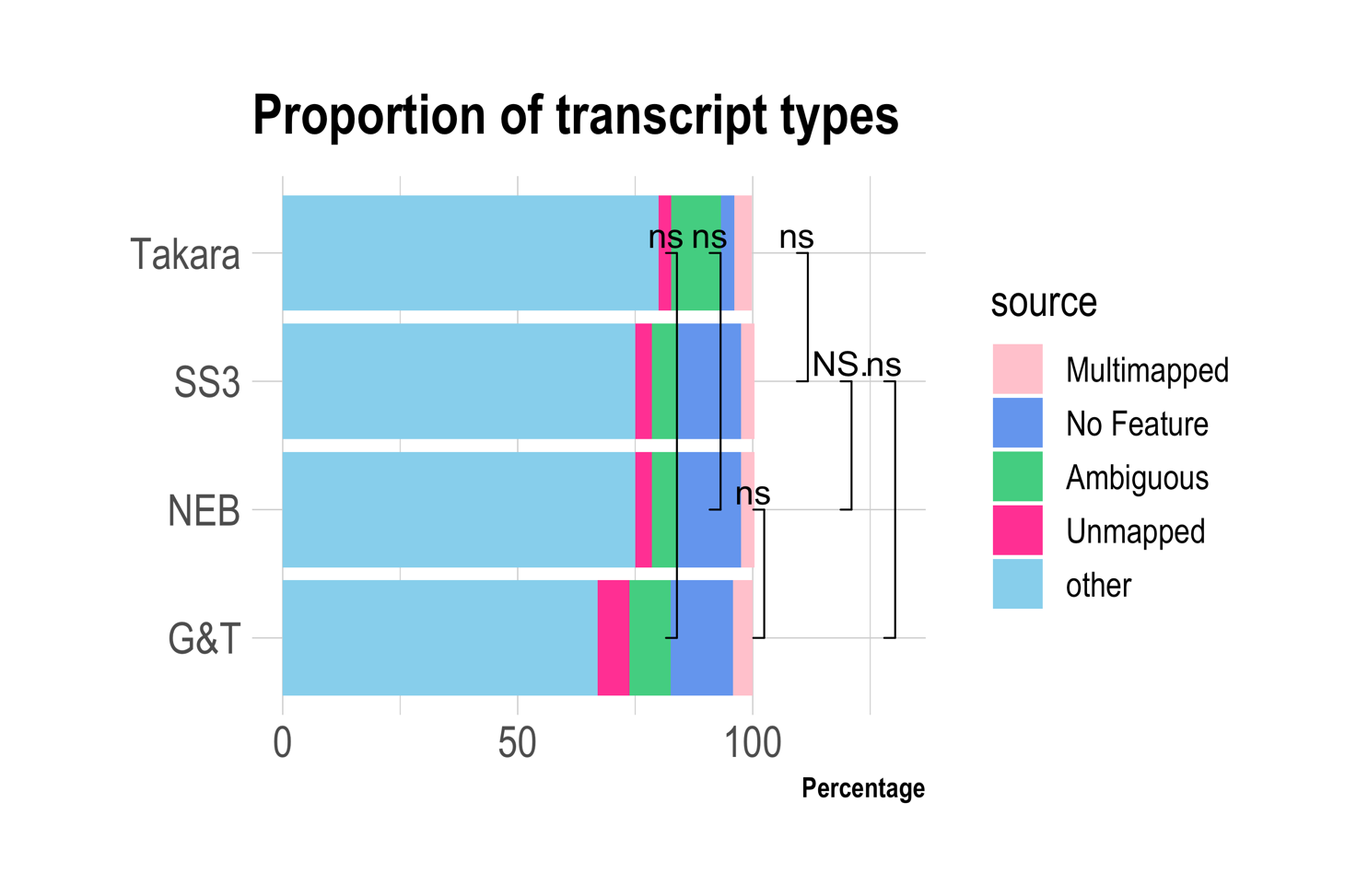
Average proportion of Multimapped, No Feature, Ambiguous and Unmapped transcripts for each protocol. Significance level cut-off: ns:p<0.05.

**Fig. S4**
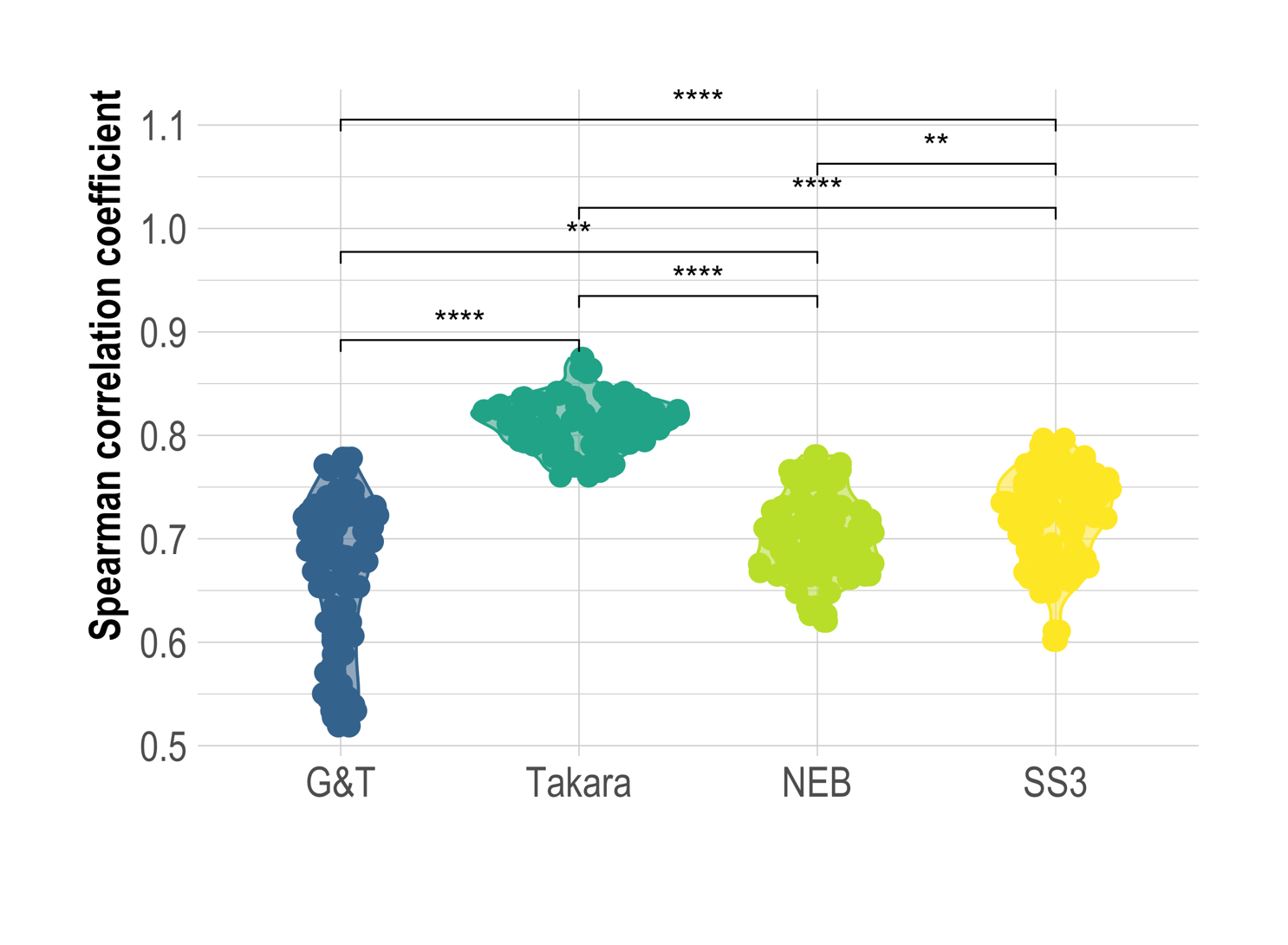
Spearman correlation coefficient (SCC) between all cells from the same protocol. The correlation was made from a geneset of the 10817 shared genes shared between the protocols (Fig. 5 D).

**Fig. S5**
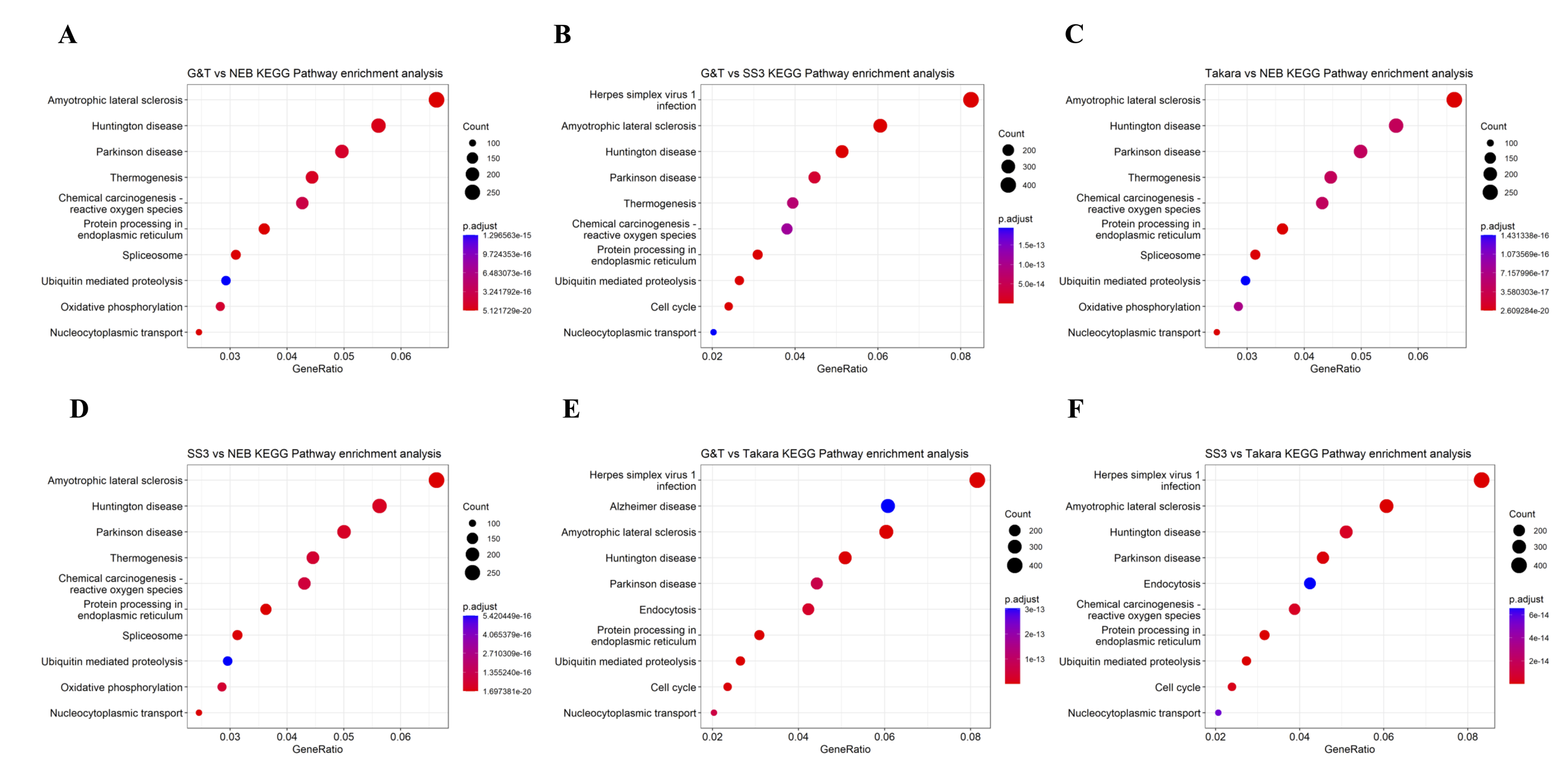


Pathway enrichment analyses of genes captured differently between cells processed by protocol A) G&T and NEB®, B) G&T and SS3, C) Takara® and NEB®, D) SS3 and NEB®, E) G&T and Takara® and F) Takara® and SS3. KEGG (46) pathway enrichment analysis was performed and visualized by using clusterProfiler (v4.4.4) (47) package from R.

**Fig. S6**

**
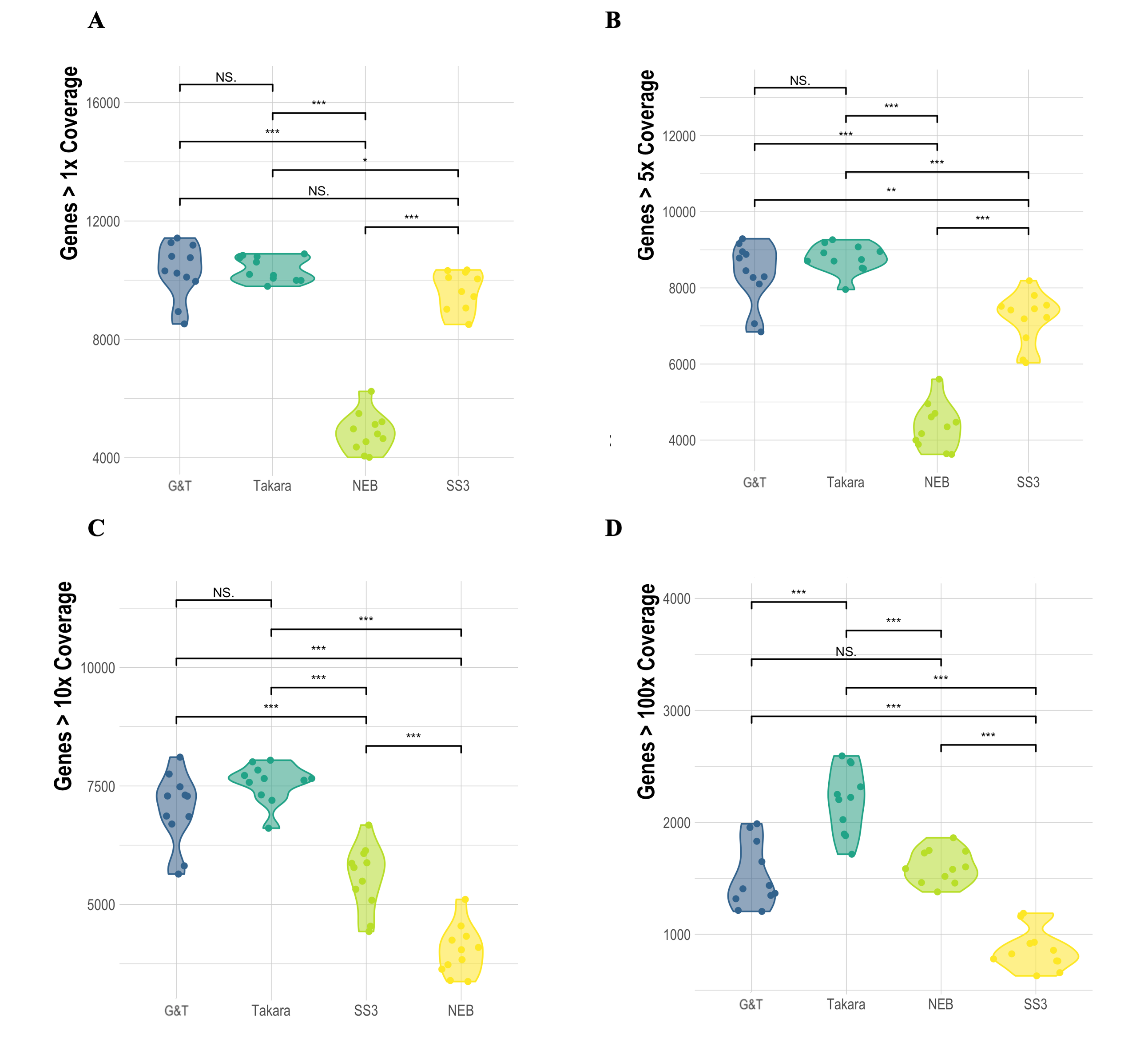
**

Calculations of coverage depth. A) Count of genes that have more than 1X coverage across coding regions.  B) Count of genes that have more than 5X coverage across coding regions.  C) Count of genes that have more than 10X coverage across coding regions D) Count of genes that have more than 100X coverage across coding regions.
